# Fibroblast growth factor 21 in dairy cows: current knowledge and potential relevance

**DOI:** 10.1186/s40104-021-00621-y

**Published:** 2021-09-14

**Authors:** Klaus Eder, Denise K. Gessner, Robert Ringseis

**Affiliations:** Institute of Animal Nutrition and Nutrition Physiology, Justus-Liebig-University Giessen, Heinrich-Buff-Ring 26-32, 35392 Giessen, Germany

**Keywords:** Dairy cow, Energy deprivation, Fibroblast growth factor 21, Metabolic adaptation, Stress

## Abstract

Fibroblast growth factor 21 (FGF21) has been identified as an important regulator of carbohydrate and lipid metabolism, which plays an important role for metabolic regulation, particularly under conditions of energy deprivation or stress conditions. Dairy cows are subjected to a negative energy balance and various kinds of stress particularly during the periparturient phase and during early lactation. It has been shown that the plasma concentration of FGF21 in dairy cows is dramatically increased at parturition and remains high during the first weeks of lactation. This finding suggests that FGF21 might exert similar functions in dairy cows than in other species, such as mice or humans. However, the role of FGF21 in dairy cows has been less investigated so far. Following a brief summary of the previous findings about the function of FGF21 in humans and mice, the present review aims to present the current state of knowledge about the role of FGF21 in dairy cows. The first part of the review deals with the tissue localization of FGF21 and with conditions leading to an upregulation of FGF21 expression in the liver of dairy cows. In the second part, the influence of nutrition on FGF21 expression and the role of FGF21 for metabolic diseases in dairy cows is addressed. In the third part, findings of exogenous FGF21 application on metabolism in dairy cows are reported. Finally, the potential relevance of FGF21 in dairy cows is discussed. It is concluded that FGF21 might be of great importance for metabolic adaptation to negative energy balance and stress conditions in dairy cows. However, further studies are needed for a better understanding of the functions of FGF21 in dairy cows.

## Introduction

Fibroblast growth factor 21 (FGF21) has been recognized as a novel metabolic regulator playing fundamental roles in carbohydrate and lipid metabolism [1]. FGF21 has been identified in humans and several animal species including rodents and non-human primates. However, most of the studies performed to explore the functions of FGF21 have been performed in rodents. Studies in rodents have shown that the expression of FGF21 is particularly increased in response to energy deprivation, during which FGF21 stimulates metabolic pathways dealing with energy mobilization, such as lipolysis, gluconeogenesis and ketogenesis [2, 3]. In addition, FGF21 is induced by various stress conditions such as environmental stress (cold), nutritional stress (fasting, malnutrition, high fat diet, obesity, amino acid deprivation) or exercise in order to cope with the energy consuming stress response by increasing availability of energy substrates [4]. In this context, FGF21 is understood as a hormone which, depending on the metabolic state, instructs the organism to reestablish homeostasis through actions on multiple tissues [5]. While the functions of FGF21 in humans and animal models, particularly mice, are increasingly understood, the knowledge about the role of FGF21 in livestock species is still limited. However, recent studies suggest that FGF21 may play also an important role for the metabolic regulation in transition dairy cows, because high-yielding dairy cows are typically experiencing energy deprivation and are exposed to various stress conditions during the early lactation phase [6–8]. Following a brief summary of the previous findings about the function of FGF21 in humans and mice, the present review aims to present the current state of knowledge about the role of FGF21 in dairy cows.

## Current knowledge about FGF21 function in humans and mice

FGF21 has first been identified and described in the year 2000 by Nishimura et al. [9]. Owing to high similarity of its amino acid sequence with that of other fibroblast growth factors, FGF21 has been classified as a novel member of the FGF family which consists of 22 members. FGF21 has been later assigned to the FGF15/19 subfamily, whose members are also referred to as endocrine FGFs, because the latter representatives, unlike other FGF members, exert their effects in an endocrine manner [10]. Nevertheless, the biological effects of FGF21 can be also mediated via autocrine and paracrine pathways [11]. The liver has been identified as the main site of FGF21 production in humans and mice [12, 13]. However, in mice, unlike in humans, FGF21 is also expressed in pancreas, testes, gastrointestinal tract, brain, skeletal muscle and brown and white adipose tissue (WAT) at lower levels [13, 14]. The biological effects of FGF21 are mediated by binding to the FGF receptor (FGFR) family of tyrosine kinases, which are comprised of seven subtypes (FGFR1b, FGFR1c, FGFR2b, FGFR2c, FGFR3b, FGFR3c, FGFR4) [15]. FGF21 binds preferentially to FGFR1c und FGFR3c subtypes [16, 17]. However, FGFR1c is the only FGFR subtype which mediates a relevant activation of FGF21 [5, 18–20]. This has been demonstrated by the finding that genetic ablation of FGFR1 causes a complete loss of FGF21 signaling in mice [21]. In the mouse, FGFR1c is the predominant subtype of the receptor in WAT, while FGFR2c is the main subtype in the liver [11]. FGFR1c is also expressed in skeletal muscle, pancreas and brain [11]. Binding of FGF21 to the FGFRs requires the presence of its co-activator β-klotho, a transmembrane protein. FGF21 binds to the FGFRs with its N-terminus and to β-klotho with its C-terminus [22, 23]. Upon binding of β-klotho/FGF21 to the FGFR1c, a downstream signaling cascade involving mitogen-activated protein kinase and AKT signaling networks is activated [24].

The first indications about biological functions of FGF21 have been provided from a cell-based screening approach by Kharitonenkov et al. [1] showing that FGF21 stimulates glucose uptake in cultured adipocytes. Over time, several other biological functions of FGF21, which are induced under specific conditions, were reported. These FGF21 functions are described below.

### Stimulation of FGF21 secretion: role of FGF21 in stress adaptation

Initial studies in mice demonstrated that energy deprivation results already within few hours in a dramatic elevation of blood FGF21 concentration, which is caused by an increased FGF21 production in the liver and to a lesser extent in other tissues, such as WAT and skeletal muscle [2, 25, 26]. Induction of FGF21 in response to energy deprivation has been proven to be caused by activation of peroxisome proliferator-activated receptor α (PPARα), a fatty acid sensing transcription factor also known as fasting regulator, in response to the increase of circulating non-esterified fatty acids (NEFA) [2, 25, 26]. In contrast to mice, an elevation of blood FGF21 concentration in humans was observed only after a longer fasting period of at least 7 d, presumably due to the lower metabolic activity compared with mice [27, 28]. Further studies revealed, that FGF21 is not only induced during energy deprivation, but also in response to various stress stimuli. A strong induction of FGF21 especially in the liver, but also in WAT, occurs in response to diverse nutritional challenges, such as protein/amino acid deprivation and feeding of high carbohydrate (glucose/fructose) or high fat (ketotic) diets [4, 29]. FGF21 production was also reported to be induced in response to cold in WAT [30] and during exercise in skeletal muscle [31, 32]. Moreover, specific kinds of cellular stress, such as endoplasmic reticulum stress (ER stress) and mitochondrial stress, were found to cause FGF21 induction in liver and/or skeletal muscle [33–35]. Elevated blood FGF21 levels have been also observed in human subjects suffering from obesity, type 2 diabetes mellitus (T2DM) and fatty liver, likely as a result of increased NEFA concentrations in blood and ER stress induction in the liver [36–38]. As a consequence of an increased FGF21 secretion in response to different kinds of stress, different metabolic responses are triggered, such as stimulation of fatty acid oxidation, gluconeogenesis and energy expenditure and inhibition of fatty acid synthesis [4, 39]. These metabolic responses are considered as adaptative mechanisms enabling the body to combat the stress condition by improving availability of energy substrates [4].

### Biochemical effects of FGF21: observations from exogenous FGF21 administration and from transgenic mice with FGF21 overexpression

To further characterize the metabolic effects of FGF21, studies, in which either recombinant (human or murine) FGF21 was administered to animal models or transgenic mice with FGF21 overexpression were used, have been conducted. In particular, studies using mouse models revealed that exogenous administration of FGF21 induces a great number of metabolic effects in both, whole body and certain tissues, which are considered as beneficial with regard to prevention or treatment of obesity and T2DM [40–42]. Several studies with mouse models of obesity and T2DM consistently showed that FGF21 administration reduces body mass, body fat mass and fat content in the liver [40, 43, 44]. The effects of FGF21 administration on body mass and body fat mass have been mainly attributed to an induction of energy expenditure, driven by an activation of the sympathetic nerve system followed by an increase in uncoupling protein-1 (UCP-1) in WAT and brown adipose tissue (BAT), along with an elevated rate of lipolysis in WAT and an improved utilization of fatty acids by peripheral tissues [11, 43, 45, 46]. The reduction of the fat content in the liver following exogenous FGF21 is caused mainly by an increased β-oxidation of fatty acids and a reduced de novo-fatty acid synthesis [11].

Administration of FGF21 to mice fed a high fat diet was found to markedly improve oral glucose tolerance and both, hepatic and peripheral insulin sensitivity and normalizes hyperinsulinemia and hypertriglyceridemia [39]. The improvement of insulin sensitivity has been explained by a stimulation of adiponectin secretion from WAT [47]. Adiponectin is an adipokine with antidiabetic and insulin sensitizing actions. Several studies have shown that FGF21 stimulates the secretion of adiponectin from adipocytes [42, 47–49]. Increased plasma concentrations of adiponectin have been consistently observed in non-human primates and humans with metabolic diseases (T2DM, obesity, non-alcoholic steatohepatitis) following treatment with various FGF21 analogs [50–53]. Adiponectin exerts its antidiabetic and insulin-sensitizing effects in part by suppressing endogenous glucose production, stimulating peripheral fatty acid uptake and enhancing catabolism of very low-density lipoproteins [11]. Normalization of hyperglycemia in diabetic mouse models was moreover reported to be caused by improving pancreatic β-cell function (due to reduction of ER stress in pancreas), reducing glycogenolysis in the liver und stimulating glucose uptake into WAT mediated by glucose transporter 1 [39, 54, 55]. In addition, a decreased preference for the intake of sugar and alcohol, but higher intake of protein as a result of FGF21 administration has been suggested to contribute to the improvement of the diabetic condition [56].

Moreover, it was shown that exogenous FGF21 improves the blood lipid profile (reduction of triglycerides and low density lipoprotein cholesterol, increase of high density lipoprotein cholesterol). Administration of FGF21 to apolipoprotein E-deficient mice reduced arteriosclerotic plaque development, likely as a consequence of a reduced hepatic cholesterol synthesis and an inhibition of inflammatory processes in vascular endothelial cells [49, 57]. Anti-inflammatory effects of exogenous FGF21 were demonstrated to be mediated by an inhibition of nuclear factor-κB signaling pathway in different tissues, particularly under pathophysiological conditions, including mouse models of fatty liver [44, 58, 59]. Furthermore, evidence has been gained that FGF21 attenuates ER stress [60, 61] and inhibits oxidative stress by stimulating nuclear factor E2 related factor-2 (Nrf2)-dependent induction of cytoprotective and antioxidative genes [62, 63]. Transgenic overexpression of FGF21 in mice was reported to increase their life span, presumably due to altering the GH/IGF-1 signaling pathway [48].

## FGF21 in dairy cows

### Expression of FGF21, β-klotho und FGFRs in tissues of dairy cows

Expression of FGF21 in cattle has first been reported by Carriquiry et al. [64]. These investigators observed that FGF21 expression in the liver of dairy cows is upregulated during the transition from late pregnancy to early lactation. Schoenberg et al. [65] were the first to investigate the longitudinal expression of FGF21 in liver, WAT, skeletal muscle and mammary gland of cows during transition from late gestation to early lactation. The authors observed that expression levels of FGF21 at late gestation were highest in the liver. Expression levels of FGF21 in WAT and mammary gland at late gestation were only 4% and 14%, respectively, of that in the liver, whereas no FGF21 expression was found in skeletal muscle. In addition, Schoenberg et al. [65] found that plasma levels of FGF21 dramatically increased during calving. While plasma levels of FGF21 were about 75 pg/mL during late gestation, FGF21 plasma levels reached a peak level of 1,600 pg/mL during calving. After calving, plasma levels of FGF21 decreased slowly, still being higher at d 56 postpartum than during late pregnancy. Hepatic expression of FGF21 during early lactation was 12-fold higher than during late pregnancy, whereas FGF21 expression level increased only marginally in WAT and even decreased in the mammary gland [65]. This indicates that the strong increase of plasma FGF21 concentration in dairy cows during calving was exclusively caused by FGF21 induction in the liver. Thus, these results in dairy cows are in line with the findings in mice and humans that the liver is the primary site of FGF21 synthesis [12, 13].

In order to identify the target tissues of FGF21 in cattle, Schoenberg et al. [65] analyzed the expression of β-klotho, the co-activator of FGF21, in different tissues of 6-month-old heifers. The highest expression of β-klotho was found in WAT, whereby the expression levels differed depending on the WAT localization; expression was most abundant in perirenal WAT, followed in decreasing order by omental WAT, mammary WAT and subcutaneous WAT. In the liver, expression level of β-klotho was only about 10% of that in perirenal WAT. Expression level of β-klotho in all tissues investigated including skeletal muscle and kidney accounted for < 10% of that determined in the liver. In dairy cows, the highest expression of β-klotho was demonstrated in the liver during early lactation, being 2.5-fold higher than in subcutaneous WAT [18]. However, as the expression of FGFR1c is nearly absent in the liver, the activity of FGF21 signaling in the liver of dairy cows during early lactation is very low [18]. In the liver of heifers, the expression of FGFR1c was also low, around 15- or 25-fold lower than in perirenal or subcutaneous adipose tissue [65]. This finding, together with the higher expression levels of β-klotho in WAT compared to liver, indicates that WAT tissue is the main target of FGF21 in cattle, while the liver plays a negligible role in FGF21 signaling.

### Induction of FGF21 in the liver of dairy cows during early lactation

In accordance with the study from Schoenberg et al. [65], several studies showed a dramatic induction of *FGF21* gene expression in the liver during early lactation [64, 66, 67], which is accompanied by a strong increase in the plasma concentration of FGF21 [68]. Based on studies in mice, it can be assumed that induction of hepatic *FGF21* gene expression is primarily caused by the energy deprivation during early lactation, which causes a stimulation of lipolysis in WAT and an increase of plasma NEFA concentration. Caixeta et al. [69] showed that an increase of plasma concentrations of NEFA, induced by intravenous infusion of an intralipid solution, caused a drastic increase of liver *FGF21* gene expression and plasma liver FGF21 concentration in dairy cows. This finding agrees with studies which demonstrated a strong relationship between plasma NEFA and FGF21 concentrations in dairy cows [65, 68]. NEFA are potent activators of PPARα causing an activation of hepatic PPARα during early lactation in dairy cows [66, 70, 71]. Hence, stimulated production of FGF21 during early lactation in dairy cows might be mediated by PPARα which is activated by increased plasma NEFA concentrations. This assumption is supported by observations in pigs, in which activation of hepatic PPARα is also accompanied by an increased expression of FGF21 [72, 73]. This clearly indicates that PPARα acts not only as a regulator of FGF21 expression in rodents, but also in different livestock species. Nonetheless, it can be assumed that, besides activation of PPARα, the occurrence of cellular stress also contributes to the induction of hepatic *FGF21* gene expression in dairy cows. Indeed, ER stress occurs in the liver of dairy cows during early lactation and has been attributed to high levels of NEFA and the presence of oxidative stress and inflammation [74, 75].

As described above, ER stress leads to the induction of *FGF21* gene expression. Gene expression of *FGF21* is upregulated by the activation of PERK and IRE, two transducers of the unfolded protein response (UPR) that are triggered by ER stress [33, 76, 77]. It is therefore highly likely that ER stress is involved in the induction of *FGF21* gene expression in the liver of cows during early lactation. Interestingly, feeding of polyphenols to dairy cows during early lactation reduces not only hepatic expression of genes of the ER stress-induced UPR, but also of FGF21 [78–80], suggesting a relationship between the occurrence of ER stress and the production of FGF21. It is well known, that an inflammatory-like condition occurs in the liver of dairy cows during early lactation, which is caused by diverse stress stimuli arising from oxidative stress, psychosocial stress, heat stress, bacterial translocation from the gastrointestinal tract, ruminal acidosis and others [7, 81, 82], and which could also contribute to FGF21 induction. In fact, a study in mice demonstrated that lipopolysacchasride (LPS) injection-induced systemic inflammation strongly stimulates hepatic *FGF21* gene expression and increases plasma FGF21 levels [83]. This observation is in line with a study from Akbar et al. [84] demonstrating that LPS injection-induced systemic inflammation in dairy cows was accompanied by an increased hepatic expression of FGF21. The work from Feingold et al. [83] indicates that FGF21 induction is a potential strategy to combat the toxicity resulting from LPS injection-induced sepsis. In the study by Yu et al. [85], administration of growth hormone (GH) was reported to increase transcription of the FGF21 gene in the liver of Angus cattle. The action of GH was shown to be mediated by signal transducer and activator of transcription 5 (STAT5), which directly binds to the FGF21 promotor. In the study of Yu et al. [85], it was additionally shown that an increased FGF21 expression inhibits GH-induced JAK2-STAT5 signaling in the liver, a mechanism considered as a negative feedback loop. It is known that blood GH concentrations are raising in dairy cows during early lactation, while the concentration of insulin like growth factor 1 (IGF-1) is decreasing due to uncoupling of the somatotropic axis [86]. Considering these observations, it appears possible that an increased FGF21 expression during early lactation could be partially also mediated by increased plasma GH concentrations. Moreover, it is possible that FGF21 contributes to the uncoupling of the somatopropic axis (reduced production of IGF-1 during early lactation. A link between FGF21 and the somatotropic axis has been demonstrated in mice and cows. Transgenic mice with overexpression of FGF21 show hallmarks of a GH resistance (increased plasma GH concentrations, reduced plasma IGF-1 concentrations) [87]. GH resistance in these mice was probably induced by a reduced hepatic concentration of phosphorylated STAT5 which mediates the action of GH on gene transcription in the liver, including the expression of IGF-1 [87]. The authors of that study suggested that the induction of a GH resistance contributes to the diverse actions of FGF21 in response to nutrient deprivation. In cows, infusion of FGF21 caused an increase of GH and a decrease of IGF-1 in plasma in comparison to untreated control cows [88]. This finding supports the indication that FGF21 could contribute to GH resistance observed in cows during early lactation.

### Influence of nutrition on FGF21 expression in the liver of dairy cows

**Fig. 1:**
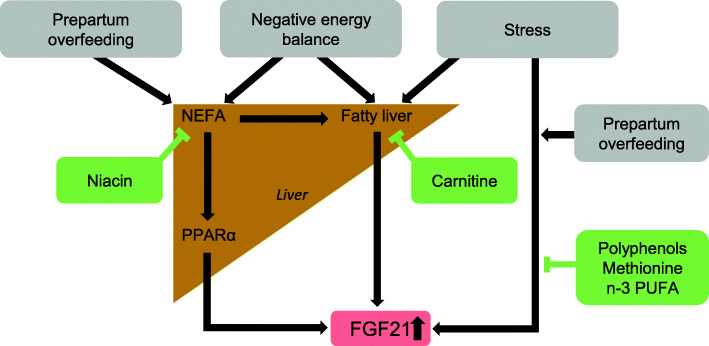
Influence of nutrition on the expression of FGF21 in the liver of dairy cows during early lactation. Negative energy balance and the occurrence of stress (ER stress, oxidative stress, inflammation) might be the main factors inducing the expression of FGF21 in the liver. Prepartum overfeeding increases hepatic expression of FGF21 in early lactation due to an increase of plasma NEFA concentrations and to an augmentation of stress as a consequence of a more severe negative energy balance. Niacin lowers FGF21 expression in dairy cows probably due to a reduction of plasma NEFA concentration. Carnitine lowers FGF21 expression in dairy cows probably due to an attenuation of fatty liver. Polyphenols, methionine and n-3 PUFA lower FGF21 expression presumably by reducing stress. For references, see text

Up to date, only few studies have investigated the influence of nutrition on FGF21 expression in dairy cows. Most of these studies were dealing with the influence of feeding intensity or body condition score prior to calving on FGF21 expression after calving. According to the majority of these studies, overfeeding during the dry period, which was associated with a higher BCS, caused increases of hepatic FGF21 expression and FGF21 blood concentrations in dairy cows during early lactation [67, 84, 89–91]. It is highly likely, that the postpartal increase of FGF21 plasma levels is caused by overfeeding-induced elevation of plasma NEFA levels during early lactation. Overfeeding during the dry period is well-known to reduce feed intake and cause a more pronounced negative energy balance during early lactation [82, 89, 90, 92]. Moreover, a higher feed intake during the prepartum period promotes the development of hepatic inflammation [82, 93]. The latter can promote the occurrence of ER stress, which itself could be a trigger of FGF21 expression [94]. Valaiti-Riboni et al. [91] reported a reduction of hepatic FGF21 expression during early lactation in dairy cows, which were overfed during the dry period, in response to supplementation of rumen-protected methionine. In this study, rumen-protected methionine was found to improve liver function, in particular the antioxidant status (increase of glutathione), and to reduce plasma levels of proinflammatory cytokines and acute phase proteins. It can be assumed, that the reduction of oxidative stress and inflammation are responsible for the decrease of hepatic FGF21 expression levels observed in cows supplemented with rumen-protected methionine. In a study from Zeitz et al. [95], a strong down-regulation of hepatic FGF21 was observed at 1 and 3 weeks postpartum in dairy cows, which were fed rumen-protected niacin during the prepartum period. In addition, the plasma NEFA levels tended to be reduced in the cows of this study, whereas no beneficial effects of rumen-protected niacin regarding occurrence of ER stress or other kinds of cellular stress could be displayed by genome-wide transcript profiling in the liver [96]. Thus, the reduced hepatic FGF21 expression in response to feeding rumen-protected niacin might be caused by decreased plasma NEFA concentrations. Akbar et al. [84] found, that supplementation of rumen-protected carnitine markedly reduces hepatic FGF21 expression in dairy cows during early lactation. Since plasma NEFA concentrations were not affected by supplemental rumen-protected carnitine, it is likely that the marked reduction of hepatic FGF21 expression was due to a lowering of hepatic triglyceride concentrations, the latter being caused by a stimulation of hepatic fatty acid β-oxidation [97]. Recent studies in mice revealed that the rate of β-oxidation plays an important role for FGF21 expression; mice with a genetic deficiency of carnitine-palmitoyl transferase (CPT)Ib in skeletal muscle or CPTII in the liver, in which β-oxidation is severely impaired, exhibit a strong upregulation of FGF21 [98, 99]. The results of the study from Lee et al. [98] suggest that upregulation of FGF21 due to CPTII deficiency is part of a systemic hormetic response, which protects mice from HFD-induced obesity and glucose intolerance. Two studies with dairy cows investigated the role of fat for hepatic FGF21 expression [64, 100]. In the study of Carriquiry et al. [64], the effect of varying the n-6/n-3 polyunsaturated fatty acid (PUFA) ratio (4.6 vs. 2.6) in the diet at late pregnancy and the beginning of lactation on the expression of FGF21 in the liver was analyzed. While the expression of FGF21 strongly increased during the transition from late pregnancy to the beginning of lactation, the n-6/n-3 PUFA ratio of the diet had no influence of FGF21 expression. In contrast, in another study, supplementation with either saturated long-chain fatty acids or fish oil caused a marked reduction of hepatic FGF21 expression postpartum, despite plasma NEFA concentrations and hepatic expression of PPARα remained unchanged [100]. A possible reason for the effect of fat supplementation on hepatic FGF21 expression might be a decrease of expression of proinflammatory genes, which was particular evident in cows supplemented with fish oil. An overview of nutritional factors influencing the expression of FGF21 is shown in Fig. 1.

### Role of FGF21 for metabolic diseases in dairy cows

A convincing relationship between FGF21 and the development of metabolic diseases in dairy cows has been established for fatty liver and ketosis. The development of fatty liver during early lactation is accompanied by an increased hepatic FGF21 expression and elevated blood FGF21 levels. Positive correlations between liver triglyceride concentration and plasma FGF21 concentration in dairy cows were documented in the studies from Schoenberg et al. [65] and Shen et al. [101]. These observations are in line with those from studies with human subjects. Like in cows, a strong correlation between liver triglyceride content and FGF21 plasma concentration has been reported in human subjects [102–104]. Based on this, the plasma FGF21 concentration has even been suggested as a surrogate marker for fatty liver in humans [105, 106]. It has been recognized that the development of a non-alcoholic fatty liver disease (NAFLD) in humans, which is frequently associated with obesity, is caused by multiple pathophysiological factors including inflammation, ER stress, oxidative stress and elevated plasma concentrations of NEFA [107]. These factors are considered to act as triggers for the induction of hepatic FGF21 expression [11, 35]. The same pathophysiological factors, such as elevated plasma NEFA levels, oxidative stress, ER stress and inflammation, can be typically found in dairy cows during early-lactation [74, 75, 108], and these factors might be involved in the development of a fatty liver and an increased production of FGF21 in the liver. Administration of FGF21 to mice was found to decrease hepatic steatosis [59]. In human subjects with NAFLD, elevated plasma FGF21 levels have been interpreted as a protective feedback signal in response to lipotoxicity arising in the liver [105]. Studies from different groups showed, that FGF21 induction prevents from hepatic lipid accumulation by stimulating AMPK-dependent lipophagy and fatty acid β-oxidation [109–111]. These findings suggest that an increased production of FGF21 in the liver of dairy cows during early lactation could also represent a means to protect against a strong hepatic lipid accumulation in the liver.

Regarding ketosis development, several studies demonstrated that the development of clinical ketosis is associated with increased hepatic expression and elevated plasma concentrations of FGF21 in dairy cows [68, 84]. Interventions resulting in increased plasma concentrations of the ketone body β-hydroxybutyric acid (BHBA) during lactation, like overfeeding during the prepartum period, were found to increase hepatic FGF21 expression [89]. In contrast to this, interventions causing a decrease of plasma BHBA concentration during lactation, as the case for supplementation with rumen-protected niacin, decreased expression von FGF21 in the liver of cows [95]. The association between BHBA und FGF21 is biologically plausible considering, that elevated plasma concentrations of ketone bodies and the development of clinical ketosis are promoted by a strong negative energy balance, which goes along with markedly elevated plasma NEFA concentrations. Thus, it is likely, that a strong negative energy balance acts as a trigger for the increased production of FGF21 in ketotic cows. Since FGF21 stimulates ketogenesis in an energy-deprived state [2, 3], it is likely, that FGF21 plays a central role for stimulating ketone body synthesis and ketosis development in dairy cows experiencing a strong negative energy balance. The well-established relationship between BHBA plasma concentrations and FGF21 production is in contradiction to a recent observation from Xu et al. [112] reporting a negative correlation between FGF21 and BHBA serum concentrations in cows. These authors postulated that low concentrations of FGF21 in plasma of cows are an indicator of ketosis development in cows [113]. However, the fact that ketosis is typically associated with increased lipid concentrations in the liver of cows, which mostly exhibit elevated blood FGF21 concentrations, argues against this postulation from Xu et al. [113].

### Effects of exogenous FGF21 on metabolism in dairy cows

The onset of lactation is associated with a strong increase in the demand of glucose for production of lactose in the mammary gland. It has been estimated, that approximately 85% of whole body glucose is transferred to the mammary gland [114]. In order to ensure a sufficient supply of the mammary gland with glucose, hormonal changes, such as reductions of insulin and the insulin-sensitizing hormone adiponectin, occur causing a status of insulin resistance [108]. Despite being important for the synthesis of lactose in the mammary gland, insulin resistance can result in unfavorable effects, such as the development of ketosis [115]. Based on studies in mice showing that exogenous FGF21 improves insulin sensitivity, a recent study investigated the effect of administration of human recombinant FGF21 both, as single injection at d 12 postpartum and chronically infused from d 14 to 23 postpartum to cows during early lactation. In this study, administration of FGF21 to the cows caused a dramatic increase of plasma FGF21 concentrations of approximately 117-fold compared to the control group [88]. As indicator of the biological activity of exogenous FGF21, the authors demonstrated an activation of the ERK1/2 signaling pathway in WAT. However, FGF21 administration did not affect plasma concentrations of insulin and adiponectin, synthesis of adiponectin and the time course of glucose and insulin concentration in response to a glucose tolerance test [18]. These observations are in contrast to those from studies reporting beneficial effects of exogenous FGF21 on insulin sensitivity in rodent models [60, 116]. The authors proposed, that the lack of effect of exogenous FGF21 on glucose concentration and insulin sensitivity in lactating cows was due to an insufficient supply of energy from feed intake resulting in a negative energy balance. Beneficial effects of exogenous FGF21 on insulin sensitivity have so far been reported only from animals experiencing excessive nutrition and obesity. This indicates, that the nutrition status is a major determinant of FGF21 actions. While feed intake, milk yield, energy balance and plasma NEFA concentration were not affected by exogenous FGF21, the hepatic triglyceride concentration was reduced by more than 50% compared to control cows [88]. This effect is beneficial considering that fatty liver development is commonly associated with the occurrence of postparturient disorders, such as subclinical and clinical ketosis, or poor reproductive performance [117]. A lowering of hepatic trigylceride content has been also documented in rodent NAFLD models following administration of FGF21 [43, 44]. In rodents, this effect was caused mainly by an inhibition of fatty acid synthesis and a stimulation of fatty acid oxidation [59, 98, 118]. In contrast, an inhibition of lipogenesis is unlikely to be causative for this effect in dairy cows, because cows generally exhibit a low rate of hepatic lipogenesis, which does not significantly contribute to fatty liver development [119]. Since the expression of genes involved in fatty acid transport, fatty acid activation, fatty acid oxidation and ketogenesis was not altered by exogenous FGF21, Caixeta et al. [88] suggested, that the reduction of hepatic triglyceride concentration was mainly due to an inhibition of lipolysis in WAT. To sum up, exogenous FGF21 in dairy cows exerts beneficial effects on the triglyceride concentration in the liver, but does not improve insulin sensitivity as could be expected from observations in rodent models. Thus, FGF21 is potentially useful as an agent to improve the health of dairy cows. However, due to the short half-life of FGF21 of about 3 h, the use of exogenous FGF21 in cows would require daily injections of FGF21. A further disadvantage of its regular use would probably be high costs.

### Potential relevance of FGF21 in dairy cows

Despite the existence of only few studies dealing with endogenous production and functions of FGF21 in dairy cows, it can be assumed, that the functions of FGF21 in dairy cows are at least similar as in other species (human, rodents). The limited number of studies available in cows shows, that, like in mice, the expression of FGF21 is stimulated particularly during a negative energy balance. Even though direct evidence is lacking, it is very likely, that FGF21 secretion is promoted under conditions of cellular stress, such as ER stress, inflammation and oxidative stress, because nutritive factors, like polyphenols and methionine, which combat these stress conditions, were shown to reduce hepatic expression of FGF21 [78, 79, 120]. Based on this, it can be assumed, that FGF21 improves the availability of energy substrates to cope with energy deprivation or stress. In mice, FGF21 has been convincingly demonstrated to stimulate lipolysis, ketogenesis, β-oxidation and gluconeogenesis [4, 39]. In contrast to mice, it is unclear which metabolic processes and to which extent are stimulated in dairy cows. As mentioned above, exogenous application of recombinant FGF21 in cows during early lactation caused a reduction of the hepatic triglyceride concentration, but had no effect on concentrations of glucose, NEFA and BHBA in plasma [18, 88]. These findings suggested, that exogenous FGF21 did not induce additional mobilization of energy by gluconeogenesis, lipolysis or ketogenesis. However, one must keep in mind, that endogenous FGF21 production in dairy cows is very high during early lactation due to the strong negative energy balance. This could possibly be a reason for the observation, that exogenous FGF21 did not induce additional effects on lipolysis and glucose homeostasis. Recent studies in diseased animal models (such as in UCP-1 transgenic mice or in CPT-1b knockout mice) have suggested that skeletal muscle-derived FGF21 may be involved in the pathogenesis of muscle atrophy [121]. It was moreover observed that in vivo overexpression of FGF21 in muscle results in an increase of autophagy and muscle loss, and that FGF21 is essential for the loss of muscle mass during fasting in mice [122]. However, as the expression of β-klotho in skeletal muscle is very low [65, 123], it is likely that FGF21 exerted its effect on muscle proteolysis rather in an indirect than in a direct manner. Although the exact relevance of this observation is not clear, a potential FGF21 function in muscle loss could be to supply of amino acids in the fasted state as substrates for gluconeogenesis. At least during the first 2 weeks of lactation, dairy cows typically do not only mobilize energy from WAT during early lactation, but also mobilize proteins from peripheral tissues, especially skeletal muscle, by proteolysis in order to provide amino acids for both, gluconeogenesis in the liver and milk protein synthesis in the mammary gland [124]. Based on the findings in mice by Oost et al. [122], it is possible, that high plasma concentrations of FGF21 during the first weeks of lactation play a role in skeletal muscle proteolysis.

Indications in mice exist, that FGF21 could play a role for female reproduction. Owen et al. [125] have observed that female transgenic mice overexpressing FGF21 are infertile. In this study, overexpression of FGF21 was accompanied by a suppression of the vasopressin-kisspeptin signaling cascade, thereby inhibiting the proestrus surge in luteinizing hormone, leading to ovulation failure and protraction of dioestrus [125]. The observation that an increased FGF21 production induced by administration of perfluorooctanoic acid (PFOA) resulted in ovulation failure and prolonged dioestrus supported a direct effect of FGF21 on reproductive function in mice [126]. Another study however showed that the impaired reproductive function in transgenic female mice with overexpression of FGF21 can be restored by feeding a high fat diet [127]. This study indicated that the infertility observed in FGF21 overexpressing mice is due to the increased energy expenditure and the caloric deficit resulting from high FGF21 levels. Although the findings in mice regarding direct effects of FGF21 on reproduction were controversial, a potential effect of FGF21 on female reproduction could be relevant to dairy cows, because it is well-known that dairy cows suffering from diseases associated with a strong negative energy balance, such as ketosis and fatty liver, typically show an impaired fertility [128]. It is not unlikely, that high concentrations of FGF21 in plasma of dairy cows suffering from negative energy balance or stress play a causative role for this phenomenon. Thus, future studies should investigate the potential relationship between FGF21 and reproductive function in dairy cows. A further interesting observation is that overexpression of FGF21 in mice stimulates bone mass loss mediated by PPARγ activation, while FGF21 knockout mice exhibit an increased bone mass compared to wild-type mice [129]. In addition, the physiological loss of bone mass during lactation was decreased in FGF21 knockout mice, likely as a consequence of a reduced bone resorption [130]. Although another study did not exert changes in bone mass in FGF21 knockout mice or in recombinant FGF21 treated mice [131], the data reported in literature provide at least some indication that high FGF21 plasma concentrations, as found in dairy cows during early lactation, promote the release of Ca^2+^ from bone mass. The mobilization of Ca^2+^ from bone mass is important to provide Ca^2+^ for milk production in the mammary gland. An insufficient mobilization of Ca^2+^ after calving is known to promote hypocalcemia, which is frequently found in high-yielding dairy cows [132]. Based on this, a relationship between FGF21 and development of hypocalcemia is not unlikely. Thus, future studies addressing this relationship might be of high relevance.

## Conclusion

**Fig. 2:**
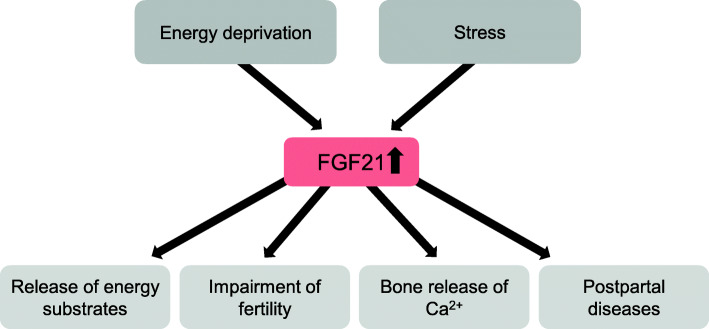
Hypothetical model of the relevance of FGF21 in dairy cows. Formation of FGF21 in dairy cows might be mainly induced by energy deprivation and stress. FGF21 might stimulate the release of energy substrates as a metabolic adaptation to combat energy deprivation and stress conditions. High concentrations of FGF21 could impair fertility and influence the release of calcium from bone which is mainly relevant during early lactation. FGF21 might also be involved in the development of postpartal diseases, such as fatty liver and ketosis. For references, see text

Studies in mice and humans have shown that FGF21 plays an important role in metabolic adaptation during states of energy deprivation or various conditions of stress. A key function of FGF21 is to increase the availability of energy substrates to cope with conditions of energy deprivation or stress [4]. During early lactation, dairy cows are not only in a negative energy balance, but experience various types of stress, including oxidative stress, ER stress or inflammation [7, 74, 82]. Therefore, it is possible that FGF21 might also play a role in metabolic adaptation of dairy cows during early lactation. This indication is supported by the finding, that the concentration of FGF21 in plasma is dramatically increased at parturition and remains at high levels during the first weeks of lactation [65]. The exact role of FGF21 in dairy cows is not known. However, the studies available so far show, that the production of FGF21 in the liver is mainly stimulated by a negative energy balance [65, 68]. Like in mice, FGF21 expression might be upregulated by PPARα, which is activated in the state of a negative energy balance by increased plasma concentrations of NEFA, which are released from WAT. There are moreover some indirect indications suggesting, that, like in mice, expression of FGF21 is increased by various stress conditions and, that inhibition of stress suppresses the expression of FGF21 in the liver [78, 79]. Like in mice and humans, FGF21 concentration in plasma of dairy cows is correlated with triglyceride concentration in the liver [65], suggesting, that FGF21 could be involved in the development of fatty liver syndrome. As FGF21 stimulates ketogenesis in the liver, concentrations of FGF21 in dairy cows might be also related to the development of ketosis during early lactation. Observations in mice indicate, that FGF21 could play a role for female fertility and bone loss during lactation [125, 129]. Therefore, it is possible, that high concentrations of FGF21 in plasma during lactation could be a reason for poor fertility, a phenomenon widely observed in high yielding dairy cows. Moreover, high plasma concentrations of FGF21 in the first days of lactation could be involved in the release of Ca^2+^ from bone required for secretion with the milk. Overall, it is likely that FGF21 not only plays an important role for metabolic adaptations in dairy cows at the onset of lactation, but might also be linked to metabolic health. A hypothetical model of the role of FGF21 in dairy cows is shown in Fig. 2. Unfortunately, there are only very few studies dealing with the potential effects and functions of FGF21 in dairy cows. In particular, further studies using recombinant FGF21 for exogenous application would be very helpful for a better understanding of the functions of FGF21 on metabolism in cattle. Improving the knowledge of the functions of FGF21 in cattle would be very useful to provide strategies for improving metabolic health in dairy cows.

## Data Availability

Not applicable.

## Abbreviations

BHBA: β-Hydroxybutyric acid
CPT: Carnitine-palmitoyl transferase
ER: Endoplasmic reticulum
FGF21: Fibroblast growth factor 21
FGFR: FGF receptor
GH: Growth hormone
LPS: Lipopolysaccharide
NAFLD: Non-alcoholic fatty liver disease
NEFA: non-esterified fatty acids
PPAR: Peroxisome proliferator-activated receptor
PUFA: Polyunsaturated fatty acid
STAT5: Signal transducer and activator of transcription 5
T2DM: Type 2 diabetes mellitus
WAT: White adipose tissue
